# Evaluation of Tumor Regulatory Genes and Apoptotic Pathways in The Cytotoxic Effect of Cytochalasin H on Malignant Human Glioma Cell Line (U87MG)

**DOI:** 10.22074/cellj.2019.5948

**Published:** 2018-11-18

**Authors:** Samaneh Heidarzadeh, Gholamreza Motalleb, Mohammad Jalil Zorriehzahra

**Affiliations:** 1 Department of Biology, Faculty of Science, University of Zabol, Zabol, Iran; 2 Department of Aquatic Animal Health and Diseases, Iranian Fisheries Science Research Institute (IFSRI), Agricultural Research, Education and Extension Organization (AREEO), Tehran, Iran

**Keywords:** Caspases, Cytochalasin H, Glioblastoma, Plasminogen Activator Urokinase, Protocadherin-10

## Abstract

**Objective:**

The aim of current study was to provide a proof-of-concept on the mechanism of *PLAU* and *PCDH10* gene expressions and caspases-3, -8, and -9 activities in the apoptotic pathway after treatment of malignant human glioma cell line (U87MG) with cytochalasin H.

**Materials and Methods:**

In the present experimental study, we have examined cytochalasin H cytotoxic activities as a new therapeutic agent on U87MG cells *in vitro* for the first time. The cells were cultured and treated with 10^-5^-10^-9^M of cytochalasin H for 24, 48 and 72 hours. The assessment of cell viability was carried out by (3-(4,5-dimethylthiazol-2-yl)- 2,5-diphenyltetrazoliumbromide (MTT) assay at 578 nm. The data are the average of three independent tests. mRNA expression changes of *PLAU* and *PCDH10* were then evaluated by quantitative reverse-transcriptase polymerase chain reaction (qRT-PCR). The fluorometric of caspases-3, -8, and -9 activities were carried out. The morphology changes in the U87MG cells were observed by fluorescence microscope.

**Results:**

MTT assay showed that cytochalasin H (10^-5^ M) inhibited the U87MG cancer cells proliferation after 48 hours. Analysis of qRT-PCR showed that the *PLAU* expression was significantly decreased in comparison with the control (P<0.05). The expression of PCDH10 also showed a significant increase when compared to the control (P<0.001). Fluorescence microscope indicated morphological changes due to apoptosis in U87MG cancer cells, after treatment with cytochalasin H (10-5M, 48 hours). The fluorometric evaluation of caspase-3, -8, and -9 activities showed no significant difference between the caspases and the control group.

**Conclusion:**

This study shows the effect of caspase-independent pathways of the programmed cell death on the U87MG cancer cell line under cytochalasin H treatment. Further studies are needed to explore the exact mechanism.

## Introduction

Glioblastoma multiforme is characterized by the code 3/9440 in the International Classification of Diseases for Oncology (ICD-O) (1). This is the most common and aggressive tumor among primary brain tumors in adults (2). Recent statistics report the incidence rate of 3.20 per 100,000 individuals for this disease (3). 

Applying the mesenchymal model, glioblastoma cells can propagate and spread to the adjacent cells. This has been proved as a limiting factor in the treatment of this tumor (4). Moreover, glioblastoma cells dramatically attack the brain parenchyma, resulting in a very poor prognosis (5, 6). 

The origin of these tumors is glial cells, composed of about 14.9% of all primary brain tumors and 56.1% of all gliomas (3). Despite the efforts carried out to improve treatment of glioma tumor, these are not curable. The conventional methods of glioblastoma treatment are surgery, radiotherapy, and chemotherapy (7). Although chemotherapy is effective in tumor treatment, the utilized drugs have side-effects. Sometimes, drug resistance causes limitations in the treatment of patients. Cytochalasins are alkaloids mycotoxins, as widely available compounds in fungi. These are extracted from an endophytic fungus, named Rhinocladiella sp, found in a Chinese medical plant, named Tripterygium. Cytochalasins target the microfilaments in the cytoskeleton (8). Cytochalasins connect to their sub-units, leading to some alterations in the cytoskeleton structure and preventing polymerization. Thus, formation of microfilaments is significantly inhibited (9). These inhibitors cause cell division by connection and interaction with the microtubule microfilament system. In addition, cellular processes are affected by cell morphology (10, 11).

Moreover, cytochalasins prevent cell transfer and create enucleated cells by penetrating the cell membrane. Additionally, cytochalasins using a variety of mechanisms affect the other biological process aspects associated with actin polymerization (12). 

Many types of cytochalasins such as A, B, C, D, E, O, and H have been identified (8), Cytochalasin H is isolated from *Paspalum scrobiculatum* Linn and affects reorganization of the cytoskeleton as an effective factor. It is a metabolite of *Phomopsis paspali*. Cytochalasin H also exerts influence on the activity of the central nervous system (13).

Apoptosis is the consequence of a planned intracellular cascade of genetically controlled stages. Caspases act an important function in the performance stage of apoptosis and they are accountable for numerous biological and morphological alterations related to the programmed cell death. Different types of caspase are identical in amino acid sequence, construction, and substrate specificity. Thusfar, 14 caspases have been recognized. Caspases have been classified based on the sequence homology, into three subclasses, including: caspase-1 subfamily (caspases-1, 4-5, 11-12 and, 13), caspase-2 subfamily (caspases-2 and-9) as well as caspase-3 subfamily (caspases-3, 6-7, 8 and-10). Caspases-2, -8 and -9 play initiator roles, while caspases-3, -6 and -7 are effectors (14, 15). 

About 840 genes have been thus far identified to be involved in the glioblastoma, study of which can lead to design glioblastoma therapeutic strategies (16). Protocadherins are the biggest subsets of cadherins in the cell adhesion molecule groups. These are mainly expressed in the nervous system (17). There have been about 70 protocadherin genes identified in the mammalian genome (18).

*PCDH10* belongs to the non-clustered protocadherins in the δ-2 protocadherin family (19). This gene is located in the chromosome 4q^28.3^ (20). *PCDH10* is considered as a tumor suppressor gene, suppressing different tumors including leukemia, lung, esophageal, colorectal and breast cancers. It is effective in cell cycle regulation and, in fact, prevents rapid growth and cell division (21).

*PLAU* gene is associated with cancer and located in the chromosome 10q^22.2^. Overexpression of urokinase plasminogen activator gene (*uPA*) and its receptor (uPAR) has been observed in the breast, bladder, lung, pancreatic, liver and colorectal cancers (22). This gene encodes serine protease, which converts plasminogen to plasmin (23). *PLAU*, as a motivatorof metastasis, encodes protein activating plasminogen urokinase, connected to the specific receptors. *PLAU* performs a key role in adjustment of the cells migration and adhesion during tissue regeneration and intracellular signaling (24). Expression of this gene in different cancers causes cell invasion and metastasis of the tumor cells to the surrounding tissues (25).

On this basis, the aim of current study was to provide a proof-of-concept on the mechanism of *PLAU* and *PCDH10* gene expressions, and caspases-3, -8, and -9 activities in the apoptotic pathway after treatment of malignant human glioma cell line (U87MG) by cytochalasin H. To our knowledge, this is the first report of cytochalasin H cytotoxic activities effect on the U87MG cells.

## Materials and Methods

### Cell culture and treatment with cytochalasin H

#### Agent treatment

Cytochalasin H was purchased from Sigma-Aldrich (USA). In all experiments, 1mg cytochalasin H was dissolved in 1ml Dimethyl Sulfoxide (DMSO, Sigma, USA) and maintained at -70°C. For cytochalasin H treatment, a relevant amount of stock solution (75 µl cytochalasin H in 15 µl DMEM medium) was prepared to the final concentrations of 10^-5^ M.

### Cell culture

In this experimental study, the malignant human glioma cell line U87MG (ATCC® HTB-14 ™) was obtained from Pasteur Institute (Iran). The cells were cultured in T25 flasks in Dulbecco’s Modified Eagle’s Medium (DMEM, Gibco-invitrogen, USA), comprising 10% fetal bovine serum (FBS, Gibco-invitrogen, USA), in 95% humidified environment at 37°C with 5% CO_2_.

### MTT assay

The 3-(4, 5-Dimethylthiazol-2-yl)-2, 5-diphenyltetrazolium (MTT) assay was employed to assess the cytotoxic impact of cytochalasin H on malignant human glioma cell line (U87MG) using Sigma-Aldrich (USA). For this, U87MG cells were placed in 96-well plates (10000 cells/ well). After 24 hours, fresh DMEM medium, containing different concentrations of cytochalasin H (10-5-10-9 M), was added at 100 µl volume per well, respectively, for 24, 48, and 72 hours. Each concentration has eight replicated wells. After incubation, the media were substituted by 100 µl of 0.5 mg/ml MTT and then the cells were further incubated at 37°C for four hours. MTT was exchanged with isopropanol and the absorbance was measured using an absorbance micro-plate reader/Elisa DNM-9602G (Madell Technology Corp, USA) at 578 nm. Furthermore, MTT assay was repeated for normal HEK cells compared to U87MG cells.

### *PLAU* and *PCDH10* quantitative reverse-transcriptase polymerase chain reaction evaluations 

For evaluating *PLAU* and *PCDH10* gene expression levels using quantitative reverse-transcriptase polymerase chain reaction (qRT-PCR) technique, U87MG cells (5×10^5^) were cultured and treated with cytochalasin H (10^-5^ M). After 24 hours, RNA was isolated by RNA extraction kit Transgen Biotech ER101-01 (China), from the U87MG cells and concentration was analyzed by nanodrop instrument (Nanodrop ND-1000 Technologies, USA). cDNA synthesis was performed using Transgen Biotech AE301-02 kit (China). Primers for amplification of PCDH10 and *PLAU* were designed using Beacon Designer, Gene Runner and Primer Express Software. The primer sequences are represented in Table 1. RTPCR program was initiated by incubating at 94°C for five minutes. This was followed by 30 cycles of 94°C, 54°C, and 72°C (30 seconds each). A last step of seven minutes (72°C) was performed. Moreover, PCR products were analyzed by agarose gel electrophoresis. qRT- PCR was carried out using ABI StepOne Real-Time PCR thermal cycler (Applied Biosystems, USA). 10 µl SYBR Green master mix, 1 µl cDNA, 1µl of forward and reverse primers (10 pmol) and 7 µl of nuclease-free water was put into each capillary tube. Each sample was performed in triplicate. The default program conditions of ABI Software were 10 minutes at 94°C (initial stage). Then, 40 cycles were carried out consisting denaturation (1 minute, 94°C), annealing and extension (70 seconds, 55°C). Melting curves were evaluated in order to confirm the specificity of PCR products.

### Morphological examination by fluorescence microscope

U87MG cells (5×10^5^) were treated with 10^-5^ M cytochalasin H for 48 hours, and subsequently collected and fixed in 80% Aston at 4°C for 20 minutes. The cells were then stained by Hoechst 33342 in dark for five minutes followed by thorough washing with phosphate- buffered saline (PBS). Finally, morphology changes in the U87MG cells were observed by fluorescence microscope (Nikon Eclipse Ti-S, USA).

### Caspase enzymatic activity assay 

The fluorometric of caspases-3, -8 and -9 activities were carried out using the NOVEX caspases kit assay (USA). This was done to quantitate the enzyme activity of caspases recognizing amino acid sequence, DEVD (for caspase-3), IETD (for caspase-8) and LEHD (for caspase-9). Briefly, U87MG cells were treated with 10^-5^ M cytochalasin H in 5% CO_2_ at 37ºC for 48 hours. Moreover, the cells (3×10^6^ per sample) were collected and added to 50 ml lysis buffer on ice for 10 minutes. Following centrifugation at 10,000 g for one minute, the lysate was collected and stored at -20°C until use. Protein concentration was assayed according to the Bradford method cytosol extract samples containing 300 µg total protein, used for caspase activity. The samples were added to 96-well plates with substrates at 37°C for two hours. The color absorbance was measured at a wave length of 405 nm in an ELISA reader (DNM-9602G, China).

### Statistical analysis

Each test was carried out in triplicate. The data are presented as mean ± SD. Student’s t test and one-way analysis of variance (ANOVA) was done to assess the significant difference through the data using IBM SPSS (IBM, USA) version 13.0. P<0.05 was considered statistically significant.

## Results

### Eeffect of cytochalasin H on the proliferation inhibition and viability of U87MG Cells

**Fig.1:**
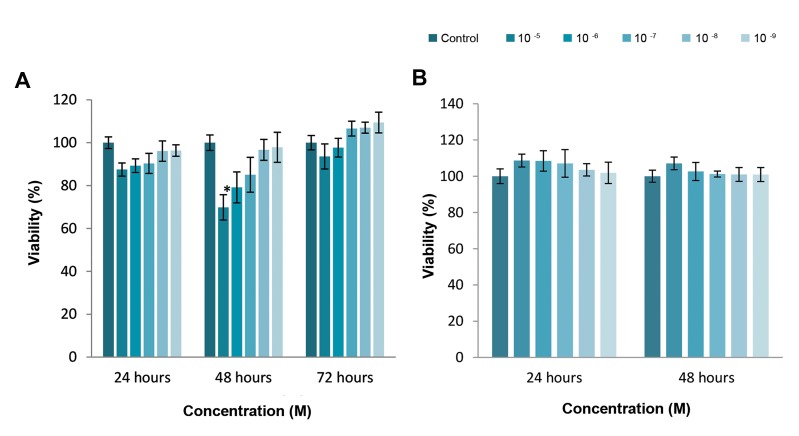
Effects of different cytochalasin H concentrations on cancer cells and normal cells compared to the control. The proliferation was determined using MTT assay. A. U87MG (cancer cells) as observed in the MTT assay during 24, 48 and 72 hours. The results are reported as means ± SD (*; P<0.05, 48 hours) and B. HEK cells (normal cells) as observed using the MTT assay after 24 and 48 hours exposure. No significant difference was observed compared to the control group.

Effect of cytochalasin H on the proliferation inhibition and viability of the U87MG cells were investigated using MTT assay. The results showed that cytochalasin H at concentration of 10^-5^ M inhibited the U87MG cancer cells proliferation for 48 hours (P<0.05), however there was no cytochalasin H toxic effects on the U87MG cancer cells after 24 and 72 hours (P>0.05, Fig .1A). Interestingly, there was not cytochalasin H toxicity effects on the normal (HEK) cell line compared to the U87MG cancer cells (P>0.05, Fig .1B). 

**Table 1:** The primer sequences applied for quantitative reverse-transcriptase polymerase chain reaction


Gene	GC	Tm (˚C)	Sequence primer (5ˊ-3ˊ)	Product size (bp)

*PCDH10*	50.0	57.9	F: TCG TGG GGA ATA TCG CTG AA	81
*PCDH10*	57.9	59.3	R: TTG AGT TGG GCA CCG TCT G	
*PLAU*	57.1	60.0	F: GGT CGC TCA AGG CTT AAC TCC	123
*PLAU*	47.6	58.8	R: CTT CAG CAA GGC AAT GTC GTT	


### Effect of cytochalasin H on the PCDH10, *PLAU* gene expressions of U87MG Cells

**Fig.2:**
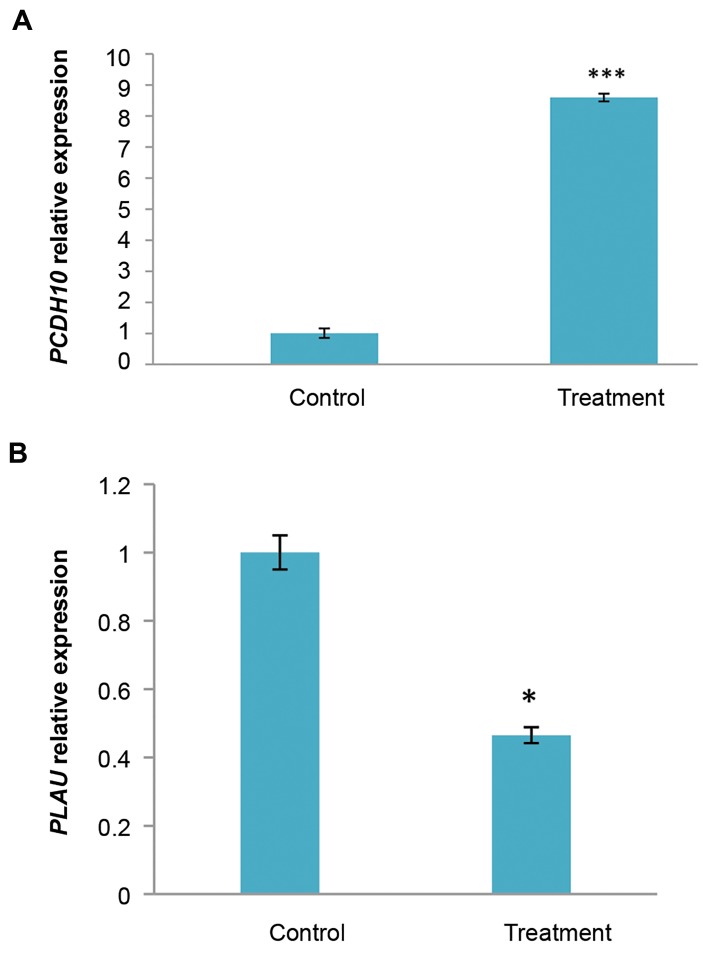
Expression level of some genes in U87MG cells after treatment with cytochalasin H (10^-5^ M) for 48 hours was evaluated by real-time polymerase chain reaction. A. PCDH10 (tumor suppressor gene) and B. *PLAU* (oncogene). The data are expressed in terms of percent of control cells as the means ± SD. ***; P<0.001 and *; P<0.05 compared to control.

The results showed that *PCDH10* gene expression was 8.59 times higher in U87MG cells treated with cytochalasinH, compared to the control. *PCDH10* gene expression wassignificantly increased (P<0.001, Fig .2A). In addition, PLAU gene expression was 2.5 times lower in U87MG cells treatedwith cytochalasin H, compared to the control. *PLAU* geneexpression was significantly decreased (P<0.05, Fig .2B).

### Assessment of caspases-3, -8 and -9 assay

**Fig.3:**
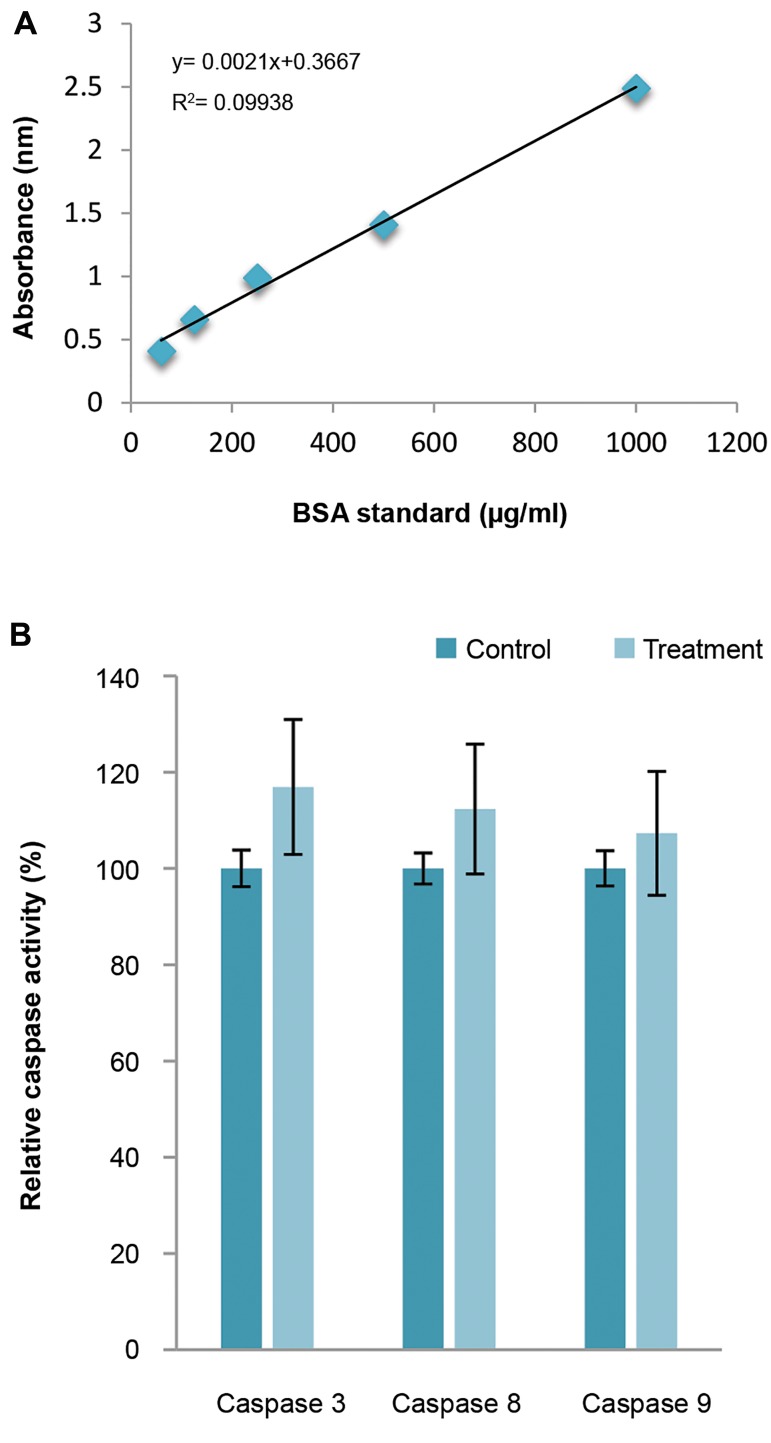
Stimulation of caspase-3, -8 and -9 Activity by cytochalasin H in U87MG Cells. A. A typical Bradford assay standard curve with samples ranging from 50 to 1000 µg/ml BSA and B. Effects of cytochalasin H on caspases-3, -8, and 9 activities. U87MG cells were treated with cytochalasin H (10^-5^ M) for 48 hours. No significant difference was determined compared to the control group.

As shown in Figure 3A, protein concentration was assassed according to Bradford standard curve containing 300 µg total protein in order to evaluate the caspase activity. 

U87MG cells were treated with cytochalasin H (10^-5^ M) for 48 hours. As shown in Figure 3B, activity of caspase-3, caspase-8 and caspase-9 were increased following cytochalasin H treatment (17, 12 and 7%, respectively), however no significant difference was observed (P>0.05). 

### Morphological observation of human malignant glioma cell line using inverted and fluorescent microscope

**Fig.4:**
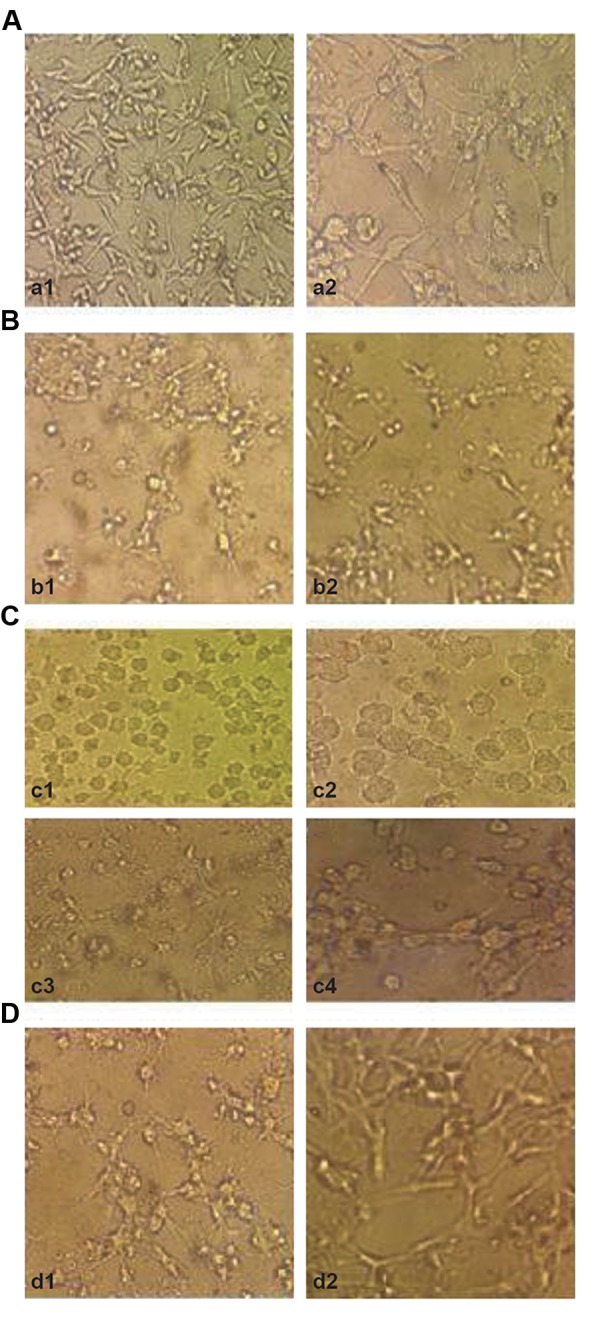
Light micrographs of the cancer cells exposed to cytochalasin H. A. Non treated U87MG cell cultures for 24, 48, 72 hours [magnifications: (a1) ×20; (a2) ×40], B. U87MG cells treated with cytochalasin H (b1) 10^-5^ M, (b2) 10^-6^ M for 24 hours [magnifications: (b1, b2) ×20], C. U87MG cell treated with cytochalasin H (c1, c2) 10^-5^ M, (c3, c4) 10^-6^ M for 48 hours [magnifications: (c1, c3) ×20, (c2, c4) ×40], and D. U87MG cell treated with cytochalasin H (d1) 10^-5^ M, (d2) 10-6 M for 72 hours [magnifications: (d1, d2) ×20].

After treatment for 24, 48 and 72 hours with 10-5 and 10^-6^ M cytochalasin H, structural changes of the cells were investigated under light microscope. Figure 4 shows the control cells with no exposure to cytochalasin H (series A), the cells exposed to cytochalasin H for 24 hours (series B), the cells exposed to cytochalasin H for 48 hours (series C) and the cells exposed to cytochalasin H for 72 hours (series D).

**Fig.5:**
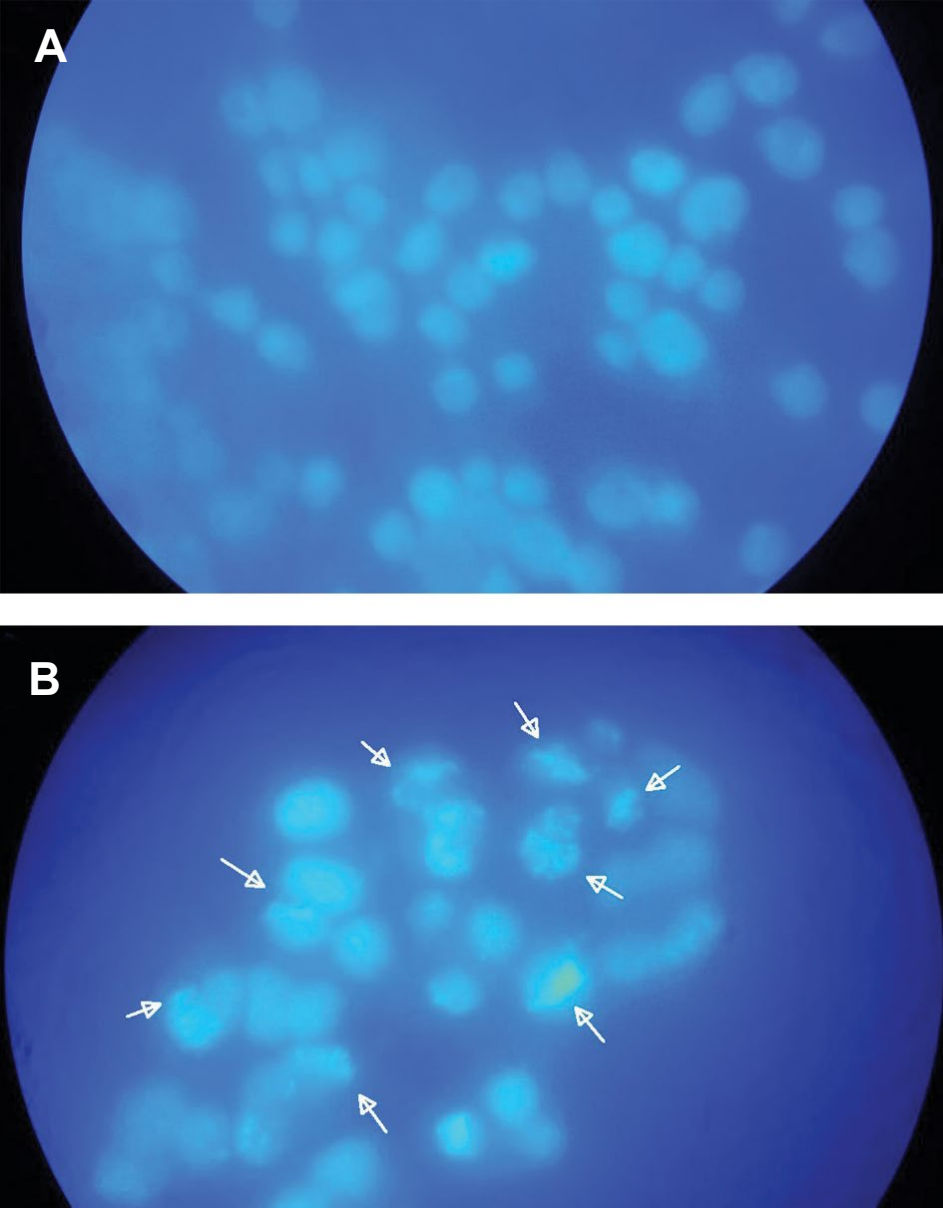
Morphological changes of U87MG cancer cells exposed to cytochalasin H (10^-5^ M) for 48 hours and imaged by fluorescence microscope. A. Illustration of the cells with normal nuclei (magnification: ×100) and B. Illustration of the cells with apoptotic nuclei (arrowheads, magnification: ×100).

After treatment for 48 hours with 10^-5^ M cytochalasin H, morphological changes were observed under fluorescence microscope. In the control group, normal nuclei were stained with a less bright blue fluorescence (Fig .5A). After cytochalasin H treatment, apoptotic cell nuclei were condensed and fragmented (Fig .5B).

## Discussion

Glioblastoma is one of the most malignant central nervous system tumors located in the brain (26), with a weak prognosis. Insufficient cytotoxic factors currently exist for curing these tumors. Cytochalasins are recognized to inhibit a number of cancer types, but the effect of cytochalasin H on glioma cells is yet unidentified.

The goal of current investigation was to evaluate special effects of cytochalasin H on the U87MG cells and apoptosis. The most important outcome of this research was that cytochalasin H inhibited human glioma U87MG cells through apoptosis in a dose- and time-dependent manner. Despite development of the standard therapeutic solutions, treatment of glioblastoma has a very bad and disappointing prognosis and it is most likely to recur (27).

The mean survival of these patients is approximately 12-15 months, which is significantly decreased in older people (27). The impact of cytochalasins on the cell morphology and performances of normal and malignant cells have been investigated *in vitro* (5, 28). Cytochalasins affect many cellular performances like cell adhesion, cell motility, secretion, drug delivery, etc. Along with chemotherapy , they induce significant clinical response in the cell systems. Cytochalasins are also considered as anti-tumor drugs for their strong feature (29).

In some studies, cytotoxic effects of cytochalasin E on the U87MG cell line (30) and impact of cytochalasin B on the U251MG, as a malignant human glioma cell line, were investigated (31). Furthermore, their impact on the inhibition of cell proliferation and growth of microfilaments were observed.

In this research, the effect of cytochalasin H (another member of cytochalasin family) on the U87MG cells was studied. Cytochalasin H significantly affects the cytoskeleton reorganization. The impact of toxicity of cytochalasin on the cancerous U87MG cell line, as well as normal HEK cells, was investigated. There was an increase of cytochalasin H toxicity in the cells treated with cytochalasine H for 48 hours. However, toxicity was statistically significant only at the concentration of 10^-5^ M. No cytochalasin H toxicity effect was observed in the cells treated with cytochalasin H for 72 hours and interestingly this important finding is in agreement with Tong et al. (31) reported that there was no difference between 72 and 96 hours after treatment of U251 cancer cell line by cytochalasin B. 

It is proposed that could be due to the deactivation of the cytochalasin H components. However, lethal effect of this compound is possibly reduced *in vitro*. Therefore, U87MG cells need more time to be reproduced. Our results showed that cytochalasin H has no toxicity effect on the normal HEK cell line, which amazingly are consistent with Trendowski (32) who reported that the cytochalasins act to preferentially injury malignant cells, as revealed by their least influences on normal epithelial and immune cells.

The obvious signs of apoptotic cells include cell nucleus condensation, chromatin and cytoplasm, loss of phosphatidylserine cell membrane, DNA fragmentation, and connection of cell membrane to the apoptotic bodies (33). It was shown that the cells were appeared in a healthy and integrated form with normal nuclei, before treatment. However, cytoplasm of the cells, treated with cytochalasin H in a concentration of 10^-5^ M, was observed in a bubbled form with concentrated and fragmented nuclei. This indicated that the cells were directed towards apoptosis.

Apoptotic pathway procedure occurs in two forms: caspase-dependent and caspase-independent pathways (34). Caspases play pivotal role in the caspase-dependent apoptosis. By classifying, caspase-8 and caspase-9 were emphasized as initiator caspases (35). However, caspase-3 was classified as an effective caspase. Caspase-8 was activated through different apoptotic pathways, but the main apoptosis induction pathway was made through the extrinsic apoptosis pathway with the help of extrinsic apoptosis induction markers of involving first apoptosis signal (FAS) (transmembrane protein) and immune cells. In the intrinsic apoptosis pathway, caspase-9 was activated by the release of cytochrome C (36). Caspase-9 led to the activation of the executive caspases (such as caspase-3), which operated on its own substrate giving rise to the apoptosis process (37).

The results obtained from this study showed that the enzyme activity of caspases was not sufficient to start the caspase-dependent apoptosis process. Moreover, statistical analysis of the caspase enzyme activity was not significant and, therefore, verified our results. 

These results were inconsistent with the findings obtained from testing the cells by fluorescent microscope. The cell nuclei were observed, condensed, and fragmented under fluorescent microscope, and this is how the cells were led towards apoptosis. So, the effect of cytochalasin H on the induction of apoptosis in the U87MG cells could probably be attributed to the caspase-independent apoptosis pathway. Interestingly, our results are consistent with Trendowski (32) who reported that cytochalasins specially injure malignant cells via actin disruption.

Previous studies showed that some types of cell deaths might occur in the absence of caspase activation. Therefore, special inhibitors of caspase could stop their activity or the activity of caspase was proposed to be no sufficient for starting the caspase-dependent apoptosis process (38, 39). As of the apparent signs of caspaseindependent apoptosis pathways are mitochondrial swelling, cytoplasmic vacuolation in the absence of caspase activation or nucleus alterations (33). 

For the first time, our results showed that cytochalasin H could successfully increase the expression of *PCDH10* in the U87MG cells which is in agreement with Hirano and Takeichi (19); Nagase et al. (20); Wolverton and Lalande (21) and Andreasen et al. (22) reported that *PCDH10* gene plays a tumor suppressor role in most tumors and high expression of *PCDH10* in the tumor cells *in vitro* significantly inhibited the proliferation and re-invasion of tumor cells to the adjacent tissues.

On the other hand, our findings showed that the cytochalasin H could successfully decrease the expression of *PLAU* in U87MG cancer cells. This is compatible with Muñoz-Cánoves et al. (40) who reported that *uPA* levels were strongly down-regulated in C_2_C1_2_ myoblast cells after treating with cytochalasin B. However, they included that this phenomenon was reversible and specific. To sum it up, these results indicate the caspaseindependent pathways (most probably through the cytoskeletal structure disruptions) of the programmed cell death in the U87MG cancer cell line under cytochalasin H treatment. However, the exact mechanisms should be further investigated.

## Conclusion

Cytochalasin H shows cytotoxic activities on U87MG cells in a dose-and time-dependent manner. More importantly, cytochalasin H induced apoptosis in glioma cells via the caspase-independent pathways (most probably through the cytoskeletal structure disruptions) together with decrease the expression of *PLAU* and increase the PCDH10 respectively. 
